# Okadaic Acid Detection through a Rapid and Sensitive Amplified Luminescent Proximity Homogeneous Assay

**DOI:** 10.3390/toxins15080501

**Published:** 2023-08-14

**Authors:** Yuan Qin, Jiayu Li, Jiani Kuang, Sicheng Shen, Xiumei Zhou, Xueqin Zhao, Biao Huang, Bingnan Han

**Affiliations:** College of Life Sciences and Medicine, Zhejiang Sci-Tech University, Hangzhou, 310018, China; jiayuli2021@163.com (J.L.); kuangjiani@icloud.com (J.K.); 12218181@zju.edu.cn (S.S.); zhouxiumei824@163.com (X.Z.); zhaoxueqin2004@163.com (X.Z.)

**Keywords:** okadaic acid, amplified luminescent proximity homogeneous assay, immunoassay, shellfish, diarrheic shellfish poisoning

## Abstract

Okadaic acid (OA), a marine biotoxin produced by microalgae, poses a significant threat to mariculture, seafood safety, and human health. The establishment of a novel, highly sensitive detection method for OA would have significant practical and scientific implications. Therefore, the purpose of this study was to develop an innovative approach for OA detection. A competitive amplified luminescent proximity homogeneous assay (AlphaLISA) was developed using the principle of specific antigen–antibody binding based on the energy transfer between chemiluminescent microspheres. The method was non-washable, sensitive, and rapid, which could detect 2 × 10^−2^–200 ng/mL of OA within 15 min, and the detection limit was 4.55 × 10^−3^ ng/mL. The average intra- and inter-assay coefficients of variation were 2.54% and 6.26%, respectively. Detection of the actual sample results exhibited a good correlation with high-performance liquid chromatography. In conclusion, a simple, rapid, sensitive, and accurate AlphaLISA method was established for detecting OA and is expected to significantly contribute to marine biotoxin research.

## 1. Introduction

Marine biotoxins are natural toxic metabolites produced by harmful algal blooms that occur due to the proliferation of microalgae such as dinoflagellates, diatoms, and cyanobacteria [1]. Although biotoxin-producing microalgae make up less than one percent of all phytoplankton, their abundance can range from a few thousand to millions of cells per liter. These biotoxins have the potential to accumulate in the flesh or digestive glands of aquatic organisms, particularly shellfish, through the food chain. This can result in adverse effects on both the aquatic environment and aquaculture industry, and can pose a significant threat to human health and safety [2,3]. The marine biotoxin exposure results in approximately 60,000 cases of poisoning worldwide annually were studied, with an overall fatality rate of 1.5% [4]. The Food and Agriculture Organization of the United Nations/World Health Organization researchers classified them into eight groups according to their chemical characteristics: okadaic acid (OA) group, azaspiracid group, cyclic imine group, saxitoxin (STX) group, brevetoxin group, domoic acid (DA) group, pectenotoxin group, and yessotoxin group [5]. They can also be categorized into diarrheic shellfish poisoning (DSP), paralytic shellfish poisoning, azaspiracid shellfish poisoning, and neurologic shellfish poisoning according to their poisoning characteristics. The OA group, also known as DSP, is widely distributed globally and poses an increasing threat to human health among these groups.

OA is the most dominant member of the OA group. OA is a polyether compound of 38-carbon fatty acids produced by various genera of *Prorocentrum* and *Dinophysis*, exhibiting lipid solubility and thermal stability, originally isolated from *Halichondria okadai Kadota* and *Halichondria melanodocia* [6,7,8,9]. In light of its lipophilicity, OA can readily accumulate in the tissues of filter-feeding marine organisms that ingest toxic algae, potentially leading to direct or indirect transmission to humans through the food chain [10,11]. Thermal stability renders the cooking temperature inadequate for its inactivation [12]. As a result, OA poisoning incidents have occurred around the world since the first reported poisoning in 1961 [13]. Therefore, OA has been used to investigate various cellular processes such as cell proliferation, apoptosis, and calcium signaling as well as nitric oxide metabolism. It has been demonstrated that OA effectively inhibits serine/threonine protein phosphatase, which causes the hyperphosphorylation of cellular proteins and dysregulation of various cellular processes, making individuals experience nausea, vomiting, diarrhea, and abdominal cramps [14]. In vitro studies have demonstrated that OA induces cytotoxicity, neurotoxicity, and embryotoxicity as well as genotoxicity, tumor promotion, and carcinogenicity in various marine organisms and even in humans [15,16]. The European Union (EU) has set the regulatory limit for OA in bivalve molluscs at 0.16 mg/kg in order to effectively control the harm caused by OA to humans [17]. Therefore, establishing an effective method for detecting OA is imperative to minimize economic losses and mitigate safety risks.

Currently, the different detection methods of OA can be broadly divided into biological, chemical, and biochemical methods. Bioassay methods were initially developed according to the toxicological effects observed in animals, tissues, or cells, including mouse bioassay (MBA) and daphnia bioassay [18,19]. The MBA was previously established as the official EU method for detecting OA, but it has gradually been replaced due to ethical concerns and the instability and limited specificity of its detection results [20]. Chemical methods of determination primarily rely on the properties of biotoxin chromatography to further the requirements in quantitative detection, such as high-performance liquid chromatography (HPLC) coupled with mass spectrometry (MS), fluorescence, and ultraviolet, as well as gas chromatography [18,21]. Currently, LC-MS/MS has been established as the official EU detection method [22]. The immunoassay technique uses the specific binding between antigen and antibody to detect the analyte, which has high specificity, high sensitivity, and wide application; thus, it is an important technical method for the detection of shellfish toxin OA [23,24]. The commonly used screening detection methods include ELISA, time-resolved fluorescence immunoassay (TRFIA), and other similar techniques; ELISA has been widely used in the study of OA. In our previous study, we established a novel OA detection method utilizing TRFIA, and the detection sensitivity and other related parameters were significantly improved over ELISA, which enhanced the detection ability of OA in environmental samples. However, its detection time was not significantly shortened compared with ELISA: both need about 1–2 h. The current methods for detecting OA have not yet achieved a balance between detection sensitivity and convenience.

AlphaLISA is an assay using photoreceptor and luminescent microspheres coated with affinity coatings as carriers. The detection principle of this method is based on the interactions between biomolecules, utilizing fluorescence resonance energy transfer luminescence and silica gel microspheres as the carrier, which has the characteristics of high sensitivity and specificity, low background noise, minimal sample requirements, low consumption, simple analytical operation, and a wide range of detectable targets [25]. AlphaLISA uses the specific binding reaction between antigen and antibody to reduce the distance between photoreceptor microspheres and luminescent microspheres to less than 200 nm, resulting in fluorescence emission through chemical reaction [26,27]. AlphaLISA employs a higher wavelength excitation light and chemical energy from the reaction to emit shorter wavelength light, resulting in significantly reduced background fluorescence and enhanced interference resistance. Compared with ELISA and chemiluminescent immunoassays, AlphaLISA avoids the necessity to clarify fractions not reacting specifically, eliminates the washing step, and reduces the assay time [28]. This versatile technology has been successfully applied for the detection of everything from proteins to peptides and a variety of small molecule compounds, with applications ranging from medicine to agriculture and food [26,27,29,30]. This study aims to establish a novel rapid and accurate AlphaLISA assay for OA, which is expected to significantly contribute to the field of marine toxin detection.

## 2. Results

### 2.1. Optimization of OA-AlphaLISA

**Figure 1:**
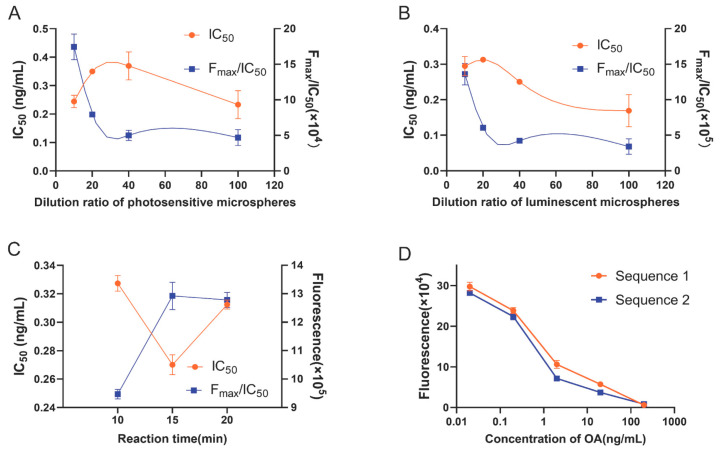
Optimization conditions of OA-AlphaLISA. (**A**) F_max_/IC_50_ values and IC_50_ values in different dilution ratios of photosensitive microspheres. (**B**) F_max_/IC_50_ values and IC_50_ values in different dilution ratios of luminescent microspheres. (**C**) F_max_/IC_50_ values and IC_50_ values in different. (**D**) Fluorescence values at different sample addition sequence.

To achieve optimal detection performance of the assay, optimization was conducted on the dilution ratio of microspheres, reaction time, and sample addition sequence. The OA standards were prepared at six different concentrations (0, 0.02, 0.2, 2, 20, and 200 ng/mL, according to the sample extraction in this study; the EU regulatory limit corresponds to 10.67 ng/mL) to construct standard curves using the control variable method for optimal condition screening. The dilution ratio of photosensitive microspheres for OA-bovine serum albumin (OA-BSA) coupling was initially screened. The dilution ratio of OA antibody-coupled luminescent microspheres was 1:40, and the reaction time was 10 min. Four alternative ratios of OA-BSA coupled photosensitive microspheres—namely, 1:10, 1:20, 1:40, and 1:100—were selected for further investigation. The results in Figure 1A reveal that a dilution ratio of 1:10 yielded smaller IC_50_ values (the half maximal inhibitory concentration) and larger F_max_/IC_50_ ratios (F: fluorescence value, F_max_: the corresponding fluorescence value at 0 concentration, maximum fluorescence value). The dilution ratio of luminescent microspheres was optimized based on the determined dilution ratio (1:10) of photosensitive microspheres. The reaction time was 10 min. Four dilution ratios of 1:10, 1:20, 1:40, and 1:100 were evaluated as alternative ratios for OA antibody-coupled luminescent microspheres. The results in Figure 1B reveal that a dilution ratio of 1:10 yielded smaller IC_50_ values and larger F_max_/IC_50_ ratios. Based on the dilution ratio of screened microspheres, the optimal reaction time was determined. Three times of 10, 15, and 20 min were selected as alternative reaction times. The results in Figure 1C indicate that low IC_50_ and high F_max_/IC_50_ were obtained at a reaction time of 15 min.

To obtain better sensitivity, two sequences were set up to optimize the sample addition sequence based on the discrepancy between the fluorescence values: sequence 1 with sequential addition of 25 μL photoreceptor microspheres, 25 μL OA standard, and 25 μL luminescent microspheres; sequence 2 with sequential addition of 25 μL luminescent microspheres, 25 μL OA standard, and 25 μL photoreceptor microspheres. As shown in Figure 1D, there was no significant difference between the two group. Sequence 1 was selected in this study.

The optimal reaction conditions obtained in this study were 1:10 dilution of photosensitive microspheres, 1:10 dilution of luminescent microspheres, and 15 min reaction time, and the spiking sequence was 25 μL photoreceptor microspheres, 25 μL OA standard, and 25 μL luminescent microspheres.

### 2.2. Identification of OA-AlphaLISA

#### 2.2.1. Sensitivity, Precision, Specificity, and Recovery Rate

**Figure 2:**
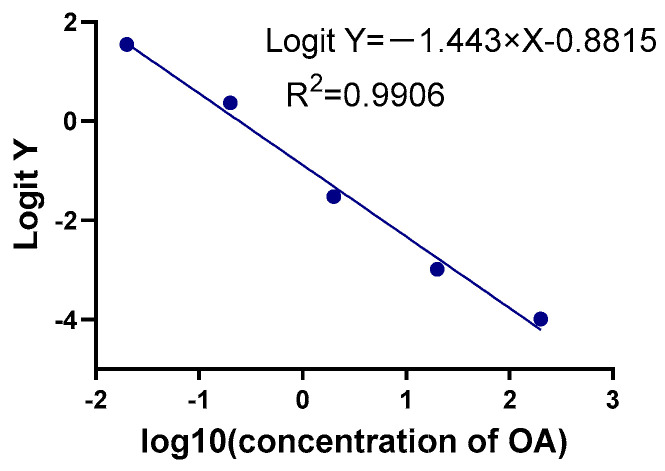
OA-AlphaLISA standard curve.

To assess the performance of the OA-AlphaLISA assay, distinct evaluations were conducted to determine its sensitivity, precision, specificity, and recovery rate. The optimized conditions were utilized to establish the standard curve of OA-AlphaLISA. Figure 2 displays the standard curve equation as Logit Y = −0.85052 − 1.43944 × X, R^2^ = 0.9908, in which Logit Y = ln(y/1 − y), y = F/F_0_, and X = lg (concentration of OA). The results exhibit that within the detection range of 2 × 10^−2^–200 ng/mL, the LOD, IC_50_, and LOQ were 4.55 × 10^−3^ ng/mL, 2.61 × 10^−1^ ng/mL, and 1.54 × 10^−1^, respectively. The variation coefficients for intra- and inter-assay batches were found to be 2.54% and 6.26%, respectively, on average. DA, STX, DTX-1, DTX-2, and MC-LR were detected using the AlphaLISA established in this work, which is summarized in Table 1. The cross-reactivity rates with DA, STX, MC-LR were all below 0.5%, which indicated that no cross-reactivity occurred, but cross-reactivity with DTX-1 and DTX-2 occurred. The results of the assay showed that cross-reactivity with DTX-1 and DTX-2 were 29.52% and 35.87%, respectively. The recovery rate of shellfish samples tested with the established AlphaLISA is shown in Table 2. The results were 95.34–104.99%, which indicated the high-level accuracy of the established assay.

#### 2.2.2. Stability

**Figure 3:**
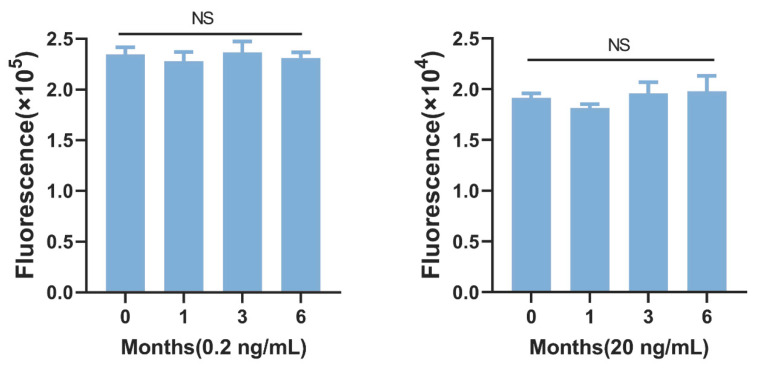
Stability of OA-AlphaLISA (NS = no significant difference).

In order to obtain stable assay results and facilitate large-scale practical detection, the stability of OA-AlphaLISA was evaluated. We stored the OA-BSA coupled to photosensitive microspheres and OA antibody coupled to the luminescent microspheres in a freezer at 4 °C, and the ratios of the detection results for 1, 3, and 6 months, with the initial values shown in Figure 3 (left: 0.2 ng/mL OA; right: 20 ng/mL). The rate of change of fluorescence values were all less than 10%, which indicated that the established OA-AlphaLISA had good stability.

### 2.3. Application of OA-AlphaLISA

**Figure 4:**
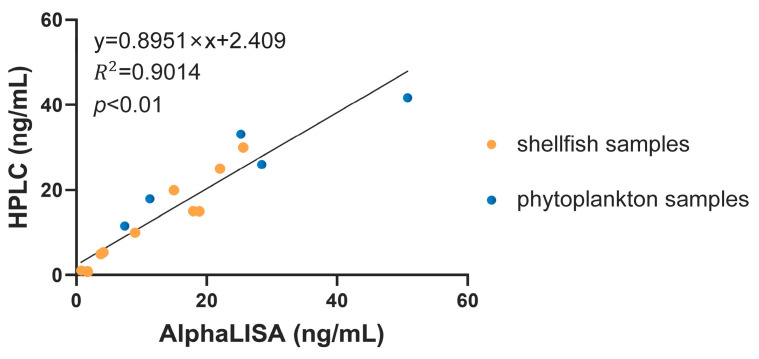
Comparison of AlphaLISA and HPLC results for the detection of samples.

Afterwards, the OA-AlphaLISA was applied to the actual samples. OA was manually added to mussel, scallop, and oyster samples and detected by OA-AlphaLISA, and the results are shown in Table S1. Simultaneous HPLC-UV was used to validate the assay results, and the correlation plot is shown in Figure 4, which suggested a good correlation (*p* < 0.01).

Natural phytoplankton were examined in order to verify the ability of the method to detect OA in natural substances. Natural phytoplankton were collected for the analysis, and the results are presented in Table S2. The correlation with HPLC was found to be good as well (Figure 4). The results of OA detection in seawater are shown in Figure S1.

## 3. Discussion

OA is a biotoxin produced by certain species of planktonic and benthic microalgae, and numerous cases of poisoning have been documented worldwide since the 1960s [31]. However, it is likely that the actual incidence of poisonings exceeds reported figures due to symptoms such as abdominal pain, diarrhea, nausea, and vomiting being commonly attributed to gastrointestinal discomfort without proper treatment. During the summer months, microalgae proliferation in areas such as the Pacific and Atlantic coasts results in a significant accumulation of OA within the aquatic ecosystem. This subsequently transfers through the food chain/web to other aquatic organisms and even humans, posing a serious threat to both ecosystems and human health [14,22,32,33]. Among aquatic organisms, bivalves have a higher capacity for accumulating OA, and mussels in particular can rapidly accumulate high levels of OA, followed by scallops and oysters [34]. At the same time, these three shellfish are also frequently consumed seafood, and the detection of OA is closely related to food safety, so these three shellfish were chosen as the subjects of this study. Due to the frequent occurrence of shellfish poisoning time in summer, the authorities have tighter control on shellfish products, and natural shellfish products enriched with shellfish toxins are difficult to obtain, so the artificial addition of shellfish toxins was chosen in this study. Furthermore, in order to further validate the ability of the method developed in this study to detect OA in natural samples, natural OA-rich phytoplankton samples collected offshore from Jiaozhou Bay, Qingdao, China were detected. Moreover, all samples were simultaneously detected with highly sensitive HPLC, which was utilized to verify the accuracy of the detected results.

In this study, a direct competition AlphaLISA method for detecting OA was developed. AlphaLISA is a highly sensitive homogeneous photoexcitation chemiluminescence immunoassay that does not require washing or separation steps and requires only a small amount of sample for detection. Moreover, for food detection, it is virtually unaffected by obstacles such as the composition of the food matrix [25]. In previous studies, AlphaLISA has been applied to the detection of T-2 toxin in food and feed with a range of 0.03–500 ng/mL, which has high sensitivity, specificity, and reproducibility [35]. This study established the OA-AlphaLISA with the detection range of 0.02–200 ng/mL, which was fully compliant with the requirements for the actual detection of OA. The IC_50_ value was determined to be 2.61 × 10^−1^ ng/mL, with a LOD of 4.55 × 10^−3^ ng/mL and LOQ of 1.54 × 10^−1^ ng/mL. The variation coefficients for intra- and inter-assay batches were found to be 2.54% and 6.26%, respectively, while the recovery rate ranged from 95.34% to 104.99%. At the same time, the OA-AlphaLISA established in this study has good stability and facilitates the implementation of large-scale practical assays. Compared with traditional biochemical methods, a significant advancement in the sensitivity and detection range was noted. ELISA is performed by coating an antigen or antibody on the polystyrene plate, then adding standard or sample extract and antibody or antigen labeled with enzyme for a competitive reaction, finally adding color development solution and termination solution for the color development reaction, and determining the concentration by absorbance. Compared to ELISA, AlphaLISA was adopted as a one-step method with the carrier of europium-containing microspheres, which shortened the incubation time, omitted the washing step, avoided the usage of enzymes, and reduced the occurrence of false positive results. Moreover, AlphaLISA is a homogeneous assay in which the components involved in the immune response are dispersed in the solution, increasing the efficiency of the reaction and thereby reducing the time. The detection range and detection sensitivity of our previously established assay OA-TRFIA were optimized by nearly five-fold compared with ELISA (detection limit 12 pg/mL, detection range 20–750 pg/mL) [24,36]. The OA-AlphaLISA established in this study reduced the detection time to one-fourth based on the application of the no-wash strategy while maintaining the same detection range and sensitivity compared with the TRFIA. The cross-reactivity rates with the three marine toxins, namely, DA, STX, and MC-LR, were less than 0.5%, indicating that no cross-reactivity occurred and the established OA-AlphaLISA had good specificity. However, cross-reactivity with OA derivatives DTX-1 and DTX-2 was observed, and the results of the assay showed that cross-reactivity with DTX-1 and DTX-2 were 29.52% and 35.87%, respectively. Similar results were also seen in other immunological methods [37,38]. In terms of molecular structure, DTX-1 contains an additional methyl group compared to OA, while DTX-2 differs from OA only in the position of a single methyl group [13]. Both compounds are analogs of OA and pose challenges for antibodies to distinguish between them completely. In addition, during shellfish metabolism, OA, DTX-1, and DTX-2 can be esterified by fatty acids with different lengths of carbon chains to form 7-O-acyl derivatives called DTX-3 [39]. DTX-3 possesses a low binding capacity for protein phosphatases but retains diarrheal effects [40]. In our study, this method was not used to assess its cross-reactivity with DTX-3, but the presence of DTX-3, which can be hydrolyzed and converted to OA in vivo, results in an increase in the concentration of OA, which should not be ignored, and will be one of the main focuses of our subsequent studies.

The OA-AlphaLISA established was also successfully applied to the examination of real samples, phytoplankton, and shellfish; the results were evaluated in comparison with HPLC that has a high sensitivity. In comparison to HPLC, AlphaLISA utilizes a relatively simple and inexpensive instrument that is easy to operate and eliminates the hydrolysis and purification steps for toxins in the sample. Specifically, sustainable development necessitates a decrease in the utilization of potent acids and bases during sample hydrolysis, as well as a reduction in the detrimental impact of chemicals on both the environment and human health. The detection method developed in this study for the detection of OA in seawater also showed good accuracy. At the same time, the assay had low sensitivity, which facilitated early detection of seawater and provided early warning for OA outbreaks. Meanwhile, it is imperative to consider the detection efficiency in actual OA detection as it pertains to food safety issues and the development of the aquaculture industry. The current on-site detection method for OA is primarily based on immunochromatography, which offers simplicity and brevity but only allows for qualitative or semi-quantitative detection with limited sensitivity. The method developed in this study employs specific antibodies for OA and is based on the principle of fluorescence resonance energy transfer, which significantly reduces reaction time while maintaining high sensitivity. This approach achieves a balance between sensitivity and convenience. With the development of new microsphere materials and coating agents and the continuous updating of antibody production technology in the future, AlphaLISA technology is more accurate and sensitive for more applications. 

There are still some shortcomings in this study. Firstly, this method exhibited cross-reactivity with OA derivatives, DTX-1 and DTX-2, which is a drawback compared to HPLC. Secondly, this study did not carry out the cross-reactivity experiment about DTX-3, but its hydrolysis in vivo leading to the increase of OA concentration should not be ignored, which will be one of the focuses of our future research. Furthermore, the sample size for detection was small and naturally occurring OA in shellfish samples was not detected. In future studies, we aim to enhance this assay by developing more specific antibodies and collecting shellfish samples that contain naturally occurring OA.

In conclusion, the AlphaLISA developed in this study for OA detection offers a simple and rapid operation with sensitive and accurate results. It can be utilized not only for shellfish and phytoplankton detection but also for seawater during harmful algae bloom formation, making it widely applicable in marine biotoxin detection.

## 4. Materials and Methods

### 4.1. Reagents and Instruments

The photoreceptor microspheres and luminescent microspheres were provided by Boshi Biological Technology (Hangzhou, China). OA-BSA, OA antibody, and OA standard were procured by the National Marine Environment Monitoring Center of China, in which OA was extracted from *Prorocentrum lima*. Assay buffer (0.05% NaN_3_, 0.01% Tween-20, 0.2% BSA, 0.9% NaCl, 50 mmol/L Tris-HCl, 20μM DTPA, pH 7.8), blocking buffer (0.2% BSA, 0.6% Tris-base, 0.05% Proclin 300, 0.9% NaCl) were made in-house by our laboratory. Proclin300 and BSA were procured from Sigma-Aldrich (Saint Louis, MO, USA). MES and Tris-base were procured from Seebio Biotechnology (Shanghai, China).

Ultrafiltration tubes of ultracel-50k (Millipore, MA, USA) were utilized for buffer exchange of OA-BSA and OA antibody, and 96-well plates (Sigma-Aldrich, Saint Louis, MO, USA) were used as reaction vessels. An ultrasonic cleaner (Suntad, Guangdong, China) was utilized to thoroughly disperse the microspheres. An electric heating incubator and a micro-oscillator were used to stimulate the immune response. A Tecan M200 (Tecan Trading AG, Männedorf, Switzerland) was used to detect the fluorescence values of the AlphaLISA reaction. Sample detection was validated with photodiode array UV-Vis detector (Shimadzu, Kyoto, Japan) and HPLC column (SilGreen, Beijing, China).

### 4.2. Conjugated Microspheres

OA-BSA and OA antibodies were coupled to photosensitive microspheres and luminescent microspheres, respectively. The OA-BSA and OA antibody were placed in ultrafiltration tubes, centrifuged eight times at 10,000 RPM (9600× *g*) for 5 min, and 300 μL of MES solution was added each time to purify the OA-BSA and OA antibody. After the last centrifugation, the MES solution was added to the ultrafiltration tubes, and they were allowed to stand for 1–2 min. The filtrate was collected by centrifugation at 3000 RPM (900× *g*) for 1 min after inverting the tube. Photosensitive microspheres and luminescent microspheres were placed in a centrifuge tube, 200 μL MES solution was added, vortexed, and sonicated until there was no obvious aggregation of microspheres, and the supernatant was discarded after centrifugation at 13,000 RPM (16,200× *g*) for 10 min. The collected OA-BSA and OA antibody were added to photosensitive microspheres and luminescent microspheres, respectively, and the MES solution was added to each of them. They underwent shaking in the dark at 37 °C. The 0.2 mol/L NaBH_4_ solution was added 10 μL after 18 to 20 h. After shaking again in the dark for 2 h, the supernatant was removed by centrifugation at 13,000 RPM (16,200× *g*) for 10 min. Then, 500 μL of blocking solution was added, vortexed, and sonicated until there was no obvious aggregation of microspheres. After blocking for 2 h, the supernatant was discarded by centrifugation at 13,000 RPM (16,200× *g*) for 10 min. Finally, 1 mL of blocking solution was added to obtain a 1 mg/mL solution of microspheres. The obtained microspheres were preserved away from light.

### 4.3. AlphaLISA Detection Protocol for OA

**Figure 5:**
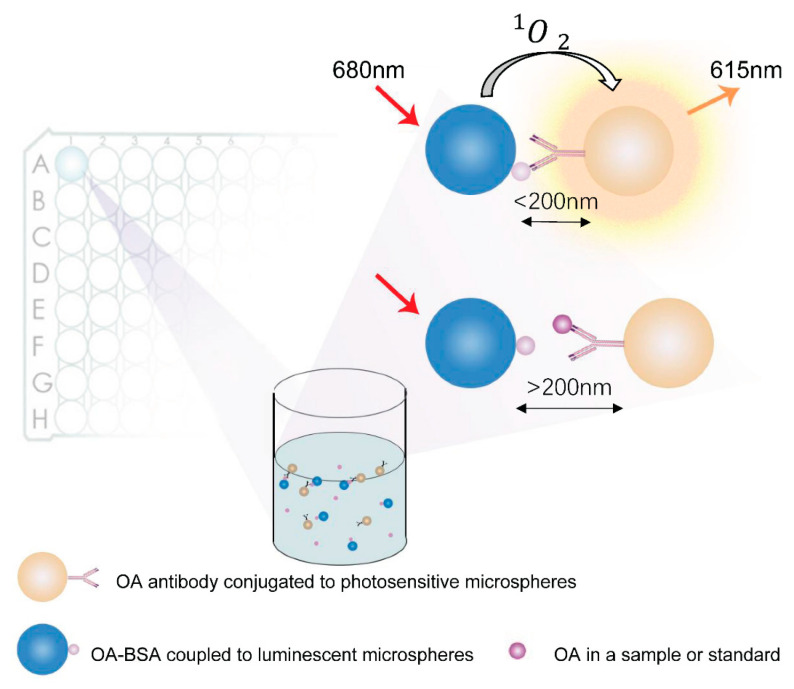
Schematic of AlphaLISA system for OA detection.

The competition method was used in this study. OA-BSA coupled to photosensitive microspheres competed with OA in the standard or sample to bind to the OA antibody coupled to the luminescent microspheres. When OA-BSA-coupled photosensitive microspheres bind with antibodies, the specific combination of antigen–antibody will close the distance between photosensitive microspheres and luminescent microspheres, so that the distance between them is less than 200 nm, which will decompose the oxygen in the surrounding environment into monomeric oxygen molecules under the irradiation of the excitation light of the instrument, and transfer the energy to Eu atoms on the luminescent microspheres to produce fluorescence. If the OA in the standard or sample is bound to the antibody, no fluorescence was produced. The detection principle is shown in Figure 5. First, OA-BSA coupled to photosensitive microspheres was added to the 96-well plate. Then, shellfish samples or OA standards and OA antibody coupled to luminescent microspheres were successively added. The 96-well plate was shaken in the dark and incubated at a temperature of 37 °C for 15 min to form antigen–antibody complexes.

### 4.4. Optimization

The dilution ratio of photosensitive microspheres and luminescent microspheres and the reaction time were optimized to enhance detection efficacy. The lower IC_50_ value and the higher F_max_/IC_50_ value were used as the selection criteria. The sample addition sequence was also optimized to obtain a higher sensitivity.

### 4.5. Method Evaluation

#### 4.5.1. Precision

The same standard was measured repeatedly four times, and its concentration mean and standard deviation were calculated to obtain the intra batch coefficient of variation. This experiment was repeated thrice to determine the coefficient of variation between batches. The coefficient of variation, which is precision was calculated by precision = standard deviation (SD)/average.

#### 4.5.2. Sensitivity

Sensitivity is the concentration level of the deviation between zero concentration standard and 2-fold SD, which requires 10 repeated determinations of the same zero concentration standard. That is the sensitivity that was calculated by the average of the F–2SD corresponding to the concentration in the standard curve. According to the Eurachem Guide, the LOD and LOQ are the concentrations corresponding to the average fluorescence values of 10 zero concentration standards minus 3- or 10-fold SD, respectively [41].

#### 4.5.3. Specificity

Cross-reactivity rates were obtained by detecting other marine biotoxins, DA, STX, and microcystin LR (MC-LR), under optimal reaction conditions. The cross-reactivity rate was calculated by cross-reactivity rate = actual concentration/theoretical concentration × 100%.

#### 4.5.4. Stability

The microspheres were placed in a freezer at 4 °C. The fluorescence values of the standards were monitored at 1, 3, and 6 months and compared with the initial values.

#### 4.5.5. Recovery Rate

The OA standard solution of the determined concentration was added to the shellfish samples of the determined concentration, and their actual concentrations were detected. Their recovery rates were calculated by the recovery rate = actual concentration/theoretical concentration × 100%.

### 4.6. Processing and Testing of Samples

The shellfish samples were homogenized, and OA was added. After adding 1 mL of methanol solution to the samples, vortex and sonication were performed. Afterwards, the supernatant was collected via a 5 min centrifugation at 8000 RPM (6200× *g*). The steps for the centrifugation and collection of the supernatant detailed above were repeated for the residue. The extracted supernatants were combined and the volume was adjusted to 3 mL [23]. Natural phytoplankton samples were taken from the offshore area of Jiaozhou Bay, Qingdao, China. The glass microfiber filters with phytoplankton were dried for 8 h at 40 °C in an oven. After weighing, they were cut into pieces with scissors and placed in 10 mL centrifuge tubes, 3 mL of methanol was added to ultrasonic extraction for 30 min, then centrifuged at 8000 RPM (6200× *g*) for 15 min and the supernatant was collected. This step was repeated twice. The supernatants were combined, concentrated, and filtered via a 0.22 μm filter membrane [42]. Seawater was collected and experiments were performed by adding standards to it.

### 4.7. HPLC Detection Protocol for OA

OA in the samples was detected using HPLC to validate the reliability of the established AlphaLISA further.

After adding 0.9 mL of methanol solution to the samples, vortexing and sonication were performed. The supernatant was extracted through centrifugation at 8000 RPM (6200× *g*) for 5 min. It was repeated by adding 0.9 mL of methanol to the residues. The extracts were combined and fixed to 2 mL. The NaOH solution of 2.5 mol/L was taken and added to the above extract, sealed, mixed, and then incubated at 70 °C for 1 h. After being cooled, the equivalent amount of HCl solution was taken in and mixed thoroughly. The mixtures were passed through a 0.22 µm filter membrane. The hydrophilic–lipophilic balance solid phase extraction column was activated with 6 mL of methanol and 6 mL of water. The filtered solution was flowed out of the extraction column at a flow rate of 1 mL/min, and then the column of solid phase extraction was washed with 20% methanol solution. No liquid was collected during this process. Finally, the eluate was performed with a methanol solution and fixed to 1 mL, collected, and passed through a 0.22 μm filter membrane again [43]. The natural phytoplankton samples were prepared identically to AlphaLISA.

The shellfish samples were detected using the C18 column and the LC-20A photodiode array UV-vis detector. H_2_O and C_2_H_3_N were chosen as the mobile phases, and the isocratic elution was carried out in the ratio of 35:65 (H_2_O: C_2_H_3_N) at a flow rate of 2 mL/min with the detection wavelength of 200 nm. The analysis time was set to 25 min, while the injection volume of the sample was fixed at 20 μL [44]. The standard curve was plotted according to the standard concentrations and peak areas, and the sample concentrations were analyzed.

### 4.8. Statistical Analysis

The correlation between HPLC and AlphaLISA was analyzed, and the IC_50_ was calculated with IBM SPSS Statistics 26 (SPSS Inc., Chicago, IL, USA). A normality test was conducted utilizing IBM SPSS Statistics 26, and a Pearson correlation test or Spearman correlation test was selected according to the results of the normality test. The results were judged to be scientifically significant at a remarkable level of *p* < 0.05.

## Figures and Tables

**Table 1:** Cross-reactivity with other marine toxins by AlphaLISA.

Compound	Cross-Reactivity (%)
DA	0.13
STX	0.02
MC-LR	0.07
DTX-1	29.52
DTX-2	35.87

**Table 2:** Recoveries of samples spiked with OA-AlphaLISA.

Sample	Spiked Concentration (ng/mL)	AlphaLIS	
		Mean Recovery ± SD (%, *n* = 3)	RSD (%)
1	0.5	95.33 ± 6.11	6.41
2	10	102.44 ± 3.65	3.56
3	50	104.98 ± 8.97	8.54

RSD: relative standard deviation.

## Data Availability

All datasets generated for this study are included in the article/supplementary material.

## Supplementary Materials

The following supporting information can be downloaded at: https://www.mdpi.com/article/10.3390/toxins15080501/s1, Figure S1: OA-AlphaLISA detection of seawater samples.; Table S1: AlphaLISA detection of shellfish samples.; Table S2: AlphaLISA detection of phytoplankton samples.
