# Supplementary material for: Okadaic Acid Detection through a Rapid and Sensitive Amplified Luminescent Proximity Homogeneous Assay
Source: Toxins (Basel). 2023 Aug 14;15(8):501. doi: 10.3390/toxins15080501 (PMC10467062; doi:10.3390/toxins15080501)
Supplement: Supplementary file 1 [file toxins-15-00501-s001.zip › toxins-2469867-supplementary.pdf]

Table S1. AlphaLISA detection of shellfish samples.

Sample category	Sample	AlphaLISA (ng/mL)	HPLC (ng/mL)
Scallop	Sample 1	0.77	1.05
	Sample 2	3.75	4.99
	Sample 3	17.96	15.04
	Sample 4	22.05	25.01
Mussel	Sample 1	1.67	0.99
	Sample 2	4.16	5.38
	Sample 3	18.89	14.97
	Sample 4	25.63	29.98
Oyster	Sample 1	1.75	0.70
	Sample 2	0.65	1.00
	Sample 3	9.01	9.99
	Sample 4	14.97	19.97

Table S2. AlphaLISA detection of phytoplankton samples.

Sample	AlphaLISA (ng/mL)	HPLC (ng/mL)
Sample 1	28.46	26.17
Sample 2	25.25	33.08
Sample 3	7.41	11.56
Sample 4	50.85	41.75
Sample 5	11.25	18.11

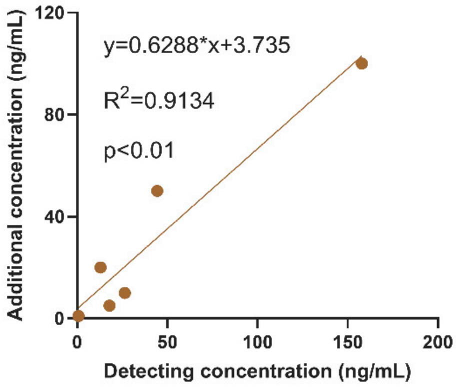

Figure S1. OA-AlphaLISA detection of seawater samples.
